# Validation of conductivity tensor imaging using giant vesicle suspensions with different ion mobilities

**DOI:** 10.1186/s12938-020-00780-5

**Published:** 2020-05-24

**Authors:** Bup Kyung Choi, Nitish Katoch, Hyung Joong Kim, Ji Ae Park, In Ok Ko, Oh In Kwon, Eung Je Woo

**Affiliations:** 1 Department of Medical Engineering, Kyung Hee University, 26, Kyungheedae-ro, Seoul, 02447 South Korea; 2 Department of Biomedical Engineering, Kyung Hee University, 1732, Deogyeong-daero, Suwon, 17104 South Korea; 3 Department of Biomedical Engineering, Kyung Hee University, 26, Kyungheedae-ro, Seoul, 02447 South Korea; 4 Division of Applied RI, Korea Institute of Radiological and Medical Science, 75, Nowonro, Seoul, 01812 South Korea; 5 Department of Mathematics, Konkuk University, 120, Neungdong-ro, Seoul, 05029 South Korea

**Keywords:** Electrical conductivity of biological tissue, Conductivity tensor imaging (CTI), Giant vesicle suspension, Magnetic resonance imaging

## Abstract

**Background:**

Electrical conductivity of a biological tissue at low frequencies can be approximately expressed as a tensor. Noting that cross-sectional imaging of a low-frequency conductivity tensor distribution inside the human body has wide clinical applications of many bioelectromagnetic phenomena, a new conductivity tensor imaging (CTI) technique has been lately developed using an MRI scanner. Since the technique is based on a few assumptions between mobility and diffusivity of ions and water molecules, experimental validations are needed before applying it to clinical studies.

**Methods:**

We designed two conductivity phantoms each with three compartments. The compartments were filled with electrolytes and/or giant vesicle suspensions. The giant vesicles were cell-like materials with thin insulating membranes. We controlled viscosity of the electrolytes and the giant vesicle suspensions to change ion mobility and therefore conductivity values. The conductivity values of the electrolytes and giant vesicle suspensions were measured using an impedance analyzer before CTI experiments. A 9.4-T research MRI scanner was used to reconstruct conductivity tensor images of the phantoms.

**Results:**

The CTI technique successfully reconstructed conductivity tensor images of the phantoms with a voxel size of $0.5\times 0.5\times 0.5\hbox { mm}^3$. The relative $L^2$ errors between the conductivity values measured by the impedance analyzer and those reconstructed by the MRI scanner was between 1.1 and 11.5.

**Conclusions:**

The accuracy of the new CTI technique was estimated to be high enough for most clinical applications. Future studies of animal models and human subjects should be pursued to show the clinical efficacy of the CTI technique.

## Background

The electrical conductivity of a biological tissue is determined not only by the concentrations and mobility of major ions in the extracellular and intracellular fluids, but also by its structural properties such as the extracellular volume fraction, extracellular matrix materials and cellular structure. At low frequencies, some biological tissues such as the white matter and muscle exhibit anisotropy in their conductivity values related with their structural properties [1].

Considering that most bioelectromagnetic phenomena occur at low frequencies below 10 kHz, for example, cross-sectional imaging of a low-frequency conductivity distribution inside the human body has been investigated for more than three decades [2]. In electrical impedance tomography (EIT), electrical currents are injected into the body and the induced voltages are measured using surface electrodes to reconstruct images of internal conductivity distributions [3, 4]. The EIT technique has been clinically used for time-difference imaging of regional lung ventilation with a temporal resolution of 25 frames/s or more [5]. Static EIT imaging, however, has not been successful so far due to technical difficulties originating from the nonlinearity and weak sensitivity between the internal conductivity distribution and the boundary current–voltage data.

For static low-frequency conductivity imaging, magnetic resonance electrical impedance tomography (MREIT) has been developed using the current-injection MRI technique [6–9]. To overcome the technical difficulties in EIT, MREIT relies on the internal data of the induced magnetic flux density distributions subject to externally injected currents along at least two different directions. MREIT, however, requires current injections of a few milliamperes during MRI scans, which hinders its clinical acceptance.

A new electrodeless conductivity tensor imaging (CTI) technique was lately proposed to produce low-frequency conductivity tensor images using an MRI scanner [10, 11]. Without injecting low-frequency currents into the human body, it may facilitate the clinical acceptance of the MRI-based low-frequency conductivity imaging method. Since the CTI technique is based on a few assumptions between mobility and diffusivity of ions and water molecules in extracellular and intracellular spaces, it should be experimentally validated before clinical application studies. Katoch et al. [11] conducted CTI imaging experiments using a conductivity phantom including a giant vesicle suspension. The giant vesicles were cell-like materials with thin insulating membranes and the giant vesicle suspension included both extracellular and intracellular spaces. However, this study was limited to a special case where the electrolytes inside and outside the giant vesicles had the same conductivity value to avoid osmotic pressure. In this paper, we further validate the CTI method using an inhomogeneous conductivity phantom with different conductivity values inside and outside the giant vesicles. To explain why such a conductivity phantom is needed, we will briefly describe the frequency dependence and direction dependence in the conductivity of a biological tissue.

### Frequency dependence of conductivity

**Fig. 1:**
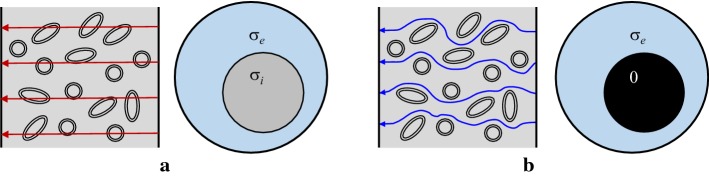
Simplified models of a biological tissue: **a** high-frequency model and** b** low-frequency model

Figure 1a, b shows simplified models of a biological tissue at high and low frequencies, respectively [1]. Each cell is assumed to be surrounded by a thin insulating membrane with negligible leakage. The thin cellular membrane is transparent to high-frequency currents, whereas it blocks dc or low-frequency currents. In Fig. 1a, therefore, the macroscopic high-frequency conductivity $\sigma _\text{H}$ of the tissue model can be expressed as

1 $$\begin{aligned} \sigma _\text{H} = \alpha \sigma _e+(1-\alpha )\sigma _i, \end{aligned}$$

where $\alpha$ is the volume fraction of the extracellular space, $\sigma _e$ is the conductivity of the extracellular space and $\sigma _i$ is the conductivity of the intracellular space. At dc or low frequencies where displacement currents are negligible, the cell behaves as a perfect insulator as shown in Fig. 1b. The low-frequency conductivity $\sigma _\text{L}$ is, therefore, expressed as

2 $$\begin{aligned} \sigma _\text{L} = \alpha \sigma _e \end{aligned}$$

since $\sigma _i=0$ in effect at low frequencies. Note that $\sigma _\text{H} > \sigma _\text{L}$ and the typical conductivity spectrum of a biological tissue shows gradually increasing conductivity values from $\sigma _\text{L}$ to $\sigma _\text{H}$ as the frequency is increased [1].

### Direction dependence of conductivity

**Fig. 2:**
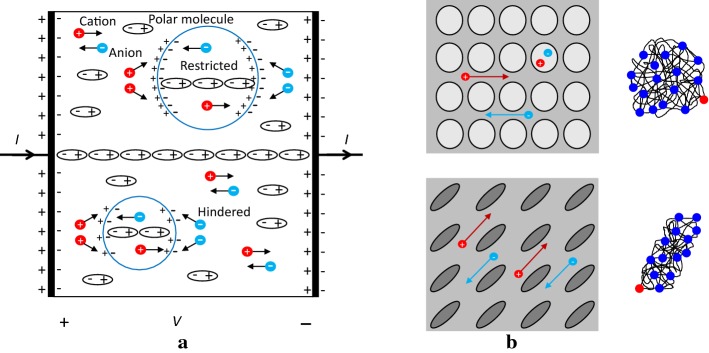
Simplified models of a microscopic environment in a biological tissue.** a** Conduction of ions, polarization of molecules and formation of charge double layers across the membranes.** b** Directional properties of ion conduction as well as diffusion affected by two different cellular structures

Some biological tissues such as the white matter and muscle exhibit anisotropy in their conductivity values at low frequencies, and the anisotropy disappears at high frequencies above 1 MHz, for example [1]. Figure 2a shows a simplified model of a microscopic environment in a biological tissue including conduction of ions, polarization of molecules such as water and formation of charged double layers across the membranes. Note that the ions inside the cells are restricted, whereas the ions outside the cells are hindered in their motions produced by an externally applied electric field.

Figure 2b shows a macroscopic view of the model in Fig. 2a, assuming two different cellular structures. At low frequencies, the ions in the extracellular space cannot penetrate the cellular membranes and must move around the cells. When the cellular structure has a directional property, the low-frequency conductivity exhibits the same directional property or anisotropy. At high frequencies, the anisotropy disappears since motions of the ions are parallel or antiparallel to the applied electric field and the cells are transparent to the resulting high-frequency currents.

The anisotropic conductivity of a biological tissue with a complicated cellular structure may have many directional components. In this paper, we express the anisotropic conductivity of a biological tissue as a conductivity tensor of $3\times 3$ matrix. Though the expression of an anisotropic conductivity as a tensor with three directional components could be an over-simplification for a real tissue with a complicated cellular structure, it provides a practically useful and mathematically manageable way of handling the tissue anisotropy.

### Brief review of CTI and its experimental validation

For a particle in a homogeneous saline solution without cells, we consider the following Einstein relation:

3 $$\begin{aligned} m=\frac{1}{k_{\text{B}}T}d, \end{aligned}$$

where *m* is mobility, *d* is diffusivity, $k_{\text{B}}$ is the Boltzmann constant and *T* is the absolute temperature. For a particle in a cellular structure, its motion is hindered and/or restricted by the structure and we may express the relation using tensors as

4 $$\begin{aligned} \mathbf{M} =\frac{1}{k_{\text{B}}T}\mathbf{D} , \end{aligned}$$

where $\mathbf{M}$ is the mobility tensor and $\mathbf{D}$ is the diffusion tensor. Since water molecules and ions exist in the same microscopic environment, we assume that they have the same directional property as

5 $$\begin{aligned} \mathbf{M} _{\text{ion}}\propto \mathbf{M} _{\text{water}},\; \mathbf{D} _{\text{ion}}\propto \mathbf{D} _{\text{water}} \text{ and } \mathbf{M} _{\text{ion}}\propto \mathbf{D} _{\text{water}}. \end{aligned}$$

The above assumption plays the key role in the CTI method where we derive a relation between the conductivity tensor and water diffusion tensor.

From (1) to (5), Sajib et al. [10] derived the following two CTI formulae. The low-frequency conductivity $\sigma _\text{L}$ of an isotropic macroscopic voxel can be expressed as

6 $$\begin{aligned} \small \sigma _\text{L} = \frac{\alpha \sigma _\text{H}}{\alpha d_\text{e}^\text{w} + (1-\alpha ) d_\text{i}^\text{w} \beta }d_\text{e}^\text{w} = \eta d_\text{e}^\text{w}, \end{aligned}$$

where $\alpha$ is the extracellular volume fraction, $\beta$ is the ion concentration ratio of intracellular and extracellular spaces, $d_\text{e}^\text{w}$ and $d_\text{i}^\text{w}$ are the extracellular and intracellular water diffusion coefficients, respectively, and $\eta$ is the position-dependent scale factor. For an anisotropic macroscopic voxel, the low-frequency conductivity tensor $\mathbf{C}$ can be expressed as

7 $$\begin{aligned} \small \mathbf{C} = \frac{\alpha \sigma _\text{H}}{\alpha {d_\text{e}^\text{w}} + (1-\alpha ) {d_\text{i}^\text{w}} \beta }\mathbf{D} _\text{e}^\text{w} = \eta \mathbf{D} _\text{e}^\text{w}, \end{aligned}$$

where $\mathbf{D} _\text{e}^\text{w}$ is the extracellular water diffusion tensor. The mean diffusivity values of the extracellular and intracellular spaces can be estimated as

8 $$\begin{aligned} d_\text{e}^\text{w}=\frac{D_{\text{e},11}^\text{w}+D_{\text{e},22}^\text{w}+D_{\text{e},33}^\text{w}}{3} \text{ and } d_\text{i}^\text{w}=\frac{D_{\text{i},11}^\text{w}+D_{\text{i},22}^\text{w}+D_{\text{i},33}^\text{w}}{3}, \end{aligned}$$

where $D_{\text{e},jj}^w$ and $D_{\text{i},jj}^w$ for $j=1,2,3$ denote the diagonal components of $\mathbf{D} _\text{e}^\text{w}$ and $\mathbf{D}_\text{i}^\text{w}$, respectively. The formulae in (6) and (7) provide a new framework for electrodeless low-frequency conductivity imaging using an MRI scanner. In the methods section, we will describe how to compute the values of the intermediate variables of $\alpha$, $\beta$, $d_\text{e}^\text{w}$, $d_\text{i}^\text{w}$ and $\mathbf{D} _\text{e}^\text{w}$.

In this paper, we validate the CTI technique using two inhomogeneous conductivity phantoms each with three compartments. The compartments were filled with electrolytes with different conductivity values or giant vesicle suspensions where the electrolytes inside and outside the giant vesicles had different conductivity values. To control the conductivity values of the giant vesicle suspensions, we adjusted viscosity of the electrolytes inside and outside the giant vesicles by adding different amounts of hyaluronic acid and polyethylene glycol solution. The conductivity values of the electrolytes and giant vesicle suspensions were measured using an impedance analyzer before CTI imaging experiments. Reconstructed conductivity tensor images using a 9.4-T research MRI scanner were compared with the conductivity values measured by the impedance analyzer for error analyses.

## Results

### Measured conductivity spectra using impedance analyzer

**Fig. 3:**
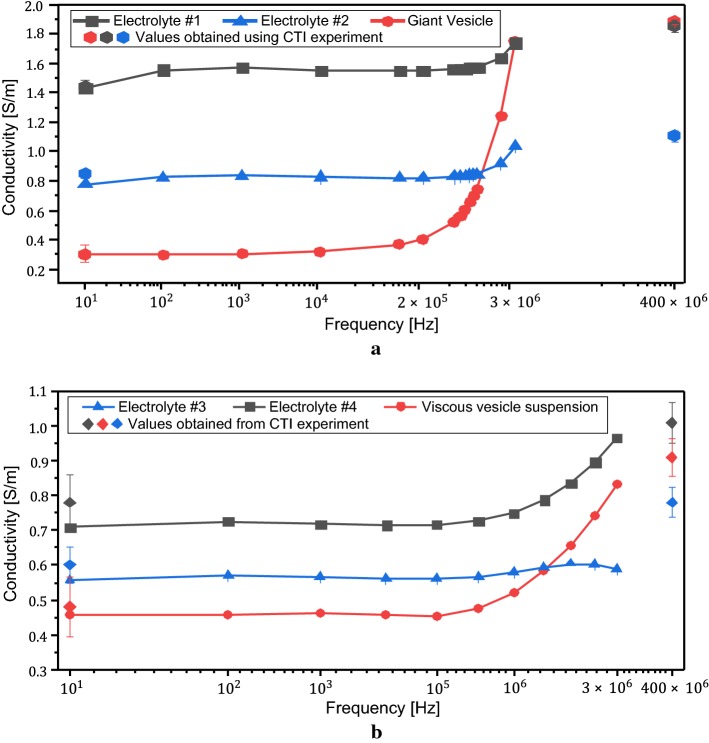
Conductivity spectra of two phantoms from 10 Hz to 3 MHz. The conductivity values from the CTI experiments were plotted as diamonds at 10 Hz and 400 MHz.** a** Conductivity spectra of the materials used in the first phantom.** b** Conductivity spectra of the materials used in the second phantom

Figure 3 shows the measured conductivity spectra of the electrolytes #1, #2, #3, #4 and the giant vesicle suspensions. The conductivity values of the electrolytes #1 and #2 at 10 Hz were 1.44 and 0.78 S/m, respectively, and increased to 1.75 and 1.04 S/m, respectively, at 3 MHz. The conductivity value of the giant vesicle suspension in the first phantom was lowest at 10 Hz and increased to about the same conductivity value of the electrolyte #1 at 3 MHz.

The electrolytes #3 and #4 in the second phantom contained the same amount of NaCl (3 g/L of distilled water). However, the electrolyte #3 showed 0.55 S/m conductivity at 10 Hz, whereas the conductivity of the electrolyte #4 at 10 Hz was 0.70 S/m. At 3 MHz, the conductivity values of the electrolytes #3 and #4 increased to 0.58 and 0.97 S/m, respectively. The smaller conductivity values of the electrolyte #3 stemmed from the reduced ion mobility caused by the increased viscosity. The giant vesicles in the second phantom were filled with the electrolyte #4 and immersed in the electrolyte #3. The conductivity values of the giant vesicle suspension in the second phantom were 0.45 S/m at 10 Hz and 0.83 S/m at 3 MHz. The value of 0.45 S/m at 10 Hz was the lowest among all measured conductivity values. At 3 MHz, the conductivity value of the giant vesicle suspension was higher than that of the electrolyte #3, but lower than that of the electrolyte #4.

### Reconstructed conductivity tensor images

**Fig. 4:**
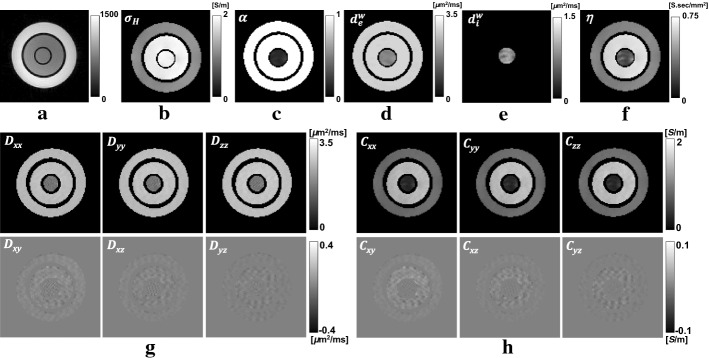
Reconstructed CTI images of the first phantom.** a**$T_2$-weighted image. Images of the** b** high-frequency conductivity ($\sigma _\text{H}$),** c** extracellular volume fraction ($\alpha$), **d** extracellular water diffusion coefficient ($d_\text{e}^\text{w}$),** e** intracellular water diffusion coefficient ($d_\text{i}^\text{w}$) and** f** scale factor ($\eta$).** g** Water diffusion tensor image. **h** Conductivity tensor image

**Fig. 5:**
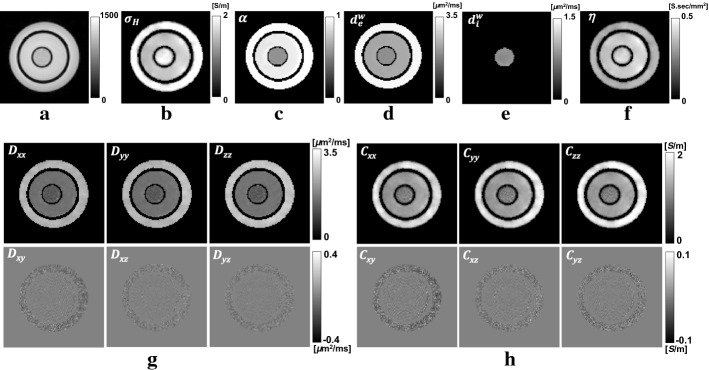
Reconstructed CTI images of the second phantom. **a**$\hbox {T}_2$-weighted image. Images of the **b** high-frequency conductivity ($\sigma _\text{H}$), **c** extracellular volume fraction ($\alpha$), **d** extracellular water diffusion coefficient ($d_\text{e}^\text{w}$), **e** intracellular water diffusion coefficient ($d_\text{i}^\text{w}$) and **f** scale factor ($\eta$). **g** Water diffusion tensor image. **h** Conductivity tensor image

Figures 4 and 5 show reconstructed CTI images of the first and second phantoms, respectively. The acquired $T_2$ images in Figs. 4a and 5a are used to define the region-of-interests (ROIs) corresponding to three different compartments in the first and second phantom, respectively. Figures 4b–f and 5b–f show the images of the intermediate variables including the high-frequency conductivity $\sigma _\text{H}$, extracellular volume fraction $\alpha$, extracellular water diffusion coefficient $d_\text{e}^\text{w}$, intracellular water diffusion coefficient $d_\text{i}^\text{w}$ and scale factor $\eta$. The conductivity tensor $\mathbf{C}$ was computed using the relation in (7). Tables 1 and 2 show the mean and standard deviation values of the intermediate variables and the diagonal components $C_{xx}$, $C_{yy}$ and $C_{zz}$ of the conductivity tensor $\mathbf{C}$ computed from all pixels in each ROI.

The values of $d_\text{e}^\text{w}$ in the electrolytes #1, #2 and #4 were $2.90\pm 0.01$, $2.85\pm 0.02$ and $2.88\pm 0.03~{\upmu \hbox {m}}^2\hbox {/ms}$, respectively, which are close to the free water diffusion coefficient [12]. In the electrolyte #3, the value of $d_\text{e}^\text{w}$ decreased to $1.97\pm ~0.02~{\upmu \hbox {m}}^2\hbox {/ms}$ due to the increased viscosity. The value of $d_\text{i}^\text{w}$ in the giant vesicle suspension of the first phantom was $0.98\pm 0.10~~\upmu {\hbox {m}}^2\hbox {/ms}$. The value of $d_\text{i}^\text{w}$ in the giant vesicle suspension of the second phantom was reduced to $0.83\pm 0.03~~{{\upmu }\hbox {m}}^2\hbox {/ms}$ since there were lesser giant vesicles in the second phantom. Since there was no giant vesicles in all electrolytes, their values of $d_\text{i}^\text{w}$ were negligible.

To compute the conductivity tensor images in Figs. 4h and 5h, the scale factors ($\eta$) were multiplied to the water diffusion tensor images in Figs. 4g and 5g, respectively. The recovered values of $C_{xx}$, $C_{yy}$ and $C_{zz}$ of the giant vesicle suspension in the first phantom were $0.29\pm 0.05$, $0.30\pm 0.07$ and $0.29\pm 0.05~\hbox {S/m}$, respectively. The corresponding values from the second phantom were $0.46\pm 0.09$, $0.47\pm 0.08$ and $0.47\pm 0.09~\hbox {S/m}$. For the electrolytes #1 to #4, the average values of the diagonal components of $\mathbf{C}$ were $1.45\pm 0.04$, $0.85\pm 0.03$, $0.59\pm 0.05$ and $0.78\pm 0.08~\hbox {S/m}$, respectively.

**Table 1:** Recovered conductivity values of the first phantom from the reconstructed CTI images

ROI	High-frequency conductivity ($\sigma _\text{H}$)	Extracellular volume fraction ($\alpha$)	Extracellular diffusion coefficient ($d_\text{e}^\text{w}$)	Intracellular diffusion coefficient ($d_\text{i}^\text{w}$)	Scale factor ($\eta$)	Low-frequency conductivity		
						$C_{xx}$	$C_{yy}$	$C_{zz}$
Giant vesicle suspension	$1.89 \pm 0.02$	$0.13\pm 0.04$	$1.93\pm 0.12$	$0.98\pm 0.10$	$0.21\pm 0.04$	$0.29\pm 0.05$	$0.30\pm 0.07$	$0.29\pm 0.05$
Electrolyte #1	$1.86\pm 0.04$	$1.00\pm 0.02$	$2.90\pm 0.01$	−	$0.64\pm 0.01$	$1.45\pm 0.04$		
Electrolyte #2	$1.11\pm 0.04$		$2.85\pm 0.02$		$0.38\pm 0.01$	$0.85\pm 0.03$		

Mean and standard deviation (SD) values of the diagonal components of the conductivity tensor $\mathbf{C}$ and the intermediate variables including the high-frequency conductivity $\sigma _\text{H}$, extracellular volume fraction $\alpha$, extracellular water diffusion coefficient $d_\text{e}^\text{w}$, intracellular water diffusion coefficient $d_\text{i}^\text{w}$ and scale factor $\eta$. The $\text{mean} \pm \text{SD}$ values were computed from all pixels within each ROI

**Table 2:** Recovered conductivity values of the second phantom from the reconstructed CTI images

ROI	High-frequency conductivity ($\sigma _\text{H}$)	Extracellular volume fraction ($\alpha$)	Extracellular diffusion coefficient ($d_\text{e}^\text{w}$)	Intracellular diffusion coefficient ($d_\text{i}^\text{w}$)	Scale factor conductivity ($\eta$)	Low-frequency		
						$C_{xx}$	$C_{yy}$	$C_{zz}$
Giant vesicle suspension	$0.92 \pm 0.05$	$0.56\pm 0.03$	$1.65\pm 0.01$	$0.83\pm 0.03$	$0.39\pm 0.05$	$0.46\pm 0.09$	$0.47\pm 0.08$	$0.47\pm 0.09$
Electrolyte #3	$0.78\pm 0.04$	$0.93\pm 0.02$	$1.97\pm 0.02$	−	$0.40\pm 0.02$	$0.59\pm 0.05$		
Electrolyte #4	$1.01\pm 0.06$	$1.01\pm 0.01$	$2.88\pm 0.03$		$0.34\pm 0.03$	$0.78\pm 0.08$		

Mean and standard deviation (SD) values of the diagonal components of the conductivity tensor $\mathbf{C}$ and the intermediate variables including the high-frequency conductivity $\sigma _\text{H}$, extracellular volume fraction $\alpha$, extracellular water diffusion coefficient $d_\text{e}^\text{w}$, intracellular water diffusion coefficient $d_\text{i}^\text{w}$ and scale factor $\eta$. The $\text{mean} \pm \text{SD}$ values were computed from all pixels within each ROI

### Recovered conductivity values

The recovered high-frequency conductivity ($\sigma _\text{H}$) values at 400 MHz (Larmor frequency at 9.4 T) were $1.86\pm 0.04$ and $1.11\pm 0.04~\hbox {S/m}$ in the electrolytes #1 and #2, respectively. For the electrolytes #3 and #4, the values of $\sigma _\text{H}$ were $0.78\pm 0.04$ and $1.01\pm 0.06\hbox { S/m}$, respectively. In the giant vesicle suspensions, the recovered $\sigma _\text{H}$ values were $1.89\pm 0.02~\hbox {S/m}$ for the first phantom and $0.92\pm 0.05~\hbox {S/m}$ for the second phantom. The values of $\alpha$ in the electrolytes #1 and #2 of the first phantom were $1.00\pm 0.02$ as expected. In the electrolyte #3, however, $\alpha$ was reduced to $0.93\pm 0.02$, whereas $\alpha$ was $1.01\pm 0.02$ in the electrolyte #4. The reduced value of $\alpha$ in the electrolyte #3 could be a result of restricted water diffusion in the viscous medium, which should have influenced the signal decay curve of the multi-b-values diffusion data. The values of $\alpha$ in the giant vesicle suspensions of the first and second phantoms were $0.13\pm 0.04$ and $0.56\pm 0.03$. This stemmed from the fact that the number of vesicles per unit volume in the first phantom was much larger than that of the second phantom. The values of $\alpha$ in the giant vesicle suspensions of the first and second phantoms were in good agreements with the microscopic images in Fig. 7c and d, respectively.

**Fig. 6:**
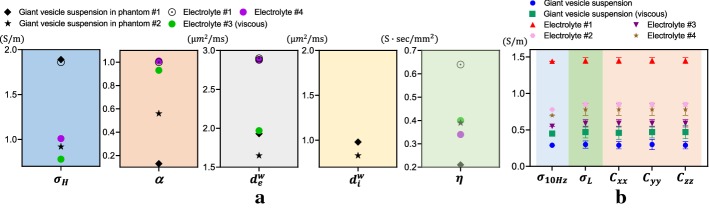
**a** Summary of the recovered values of $\sigma _\text{H}$, $\alpha$, $d_\text{e}^\text{w}$, $d_\text{i}^\text{w}$ and $\eta$. **b** Comparison of $\sigma _\text{L}=\frac{C_{xx}+C_{yy}+C_{zz}}{3}$ in the giant vesicle suspensions with their conductivity values measured at 10 Hz ($\sigma _{10\text{Hz}}$) using the impedance analyzer

Figure 6a summarizes the values of the reconstructed intermediate variables of the CTI images of the first and second phantoms. Note that the low-frequency conductivity $\sigma _\text{L}=\frac{C_{xx}+C_{yy}+C_{zz}}{3}$ values were always smaller than the high-frequency conductivity $\sigma _\text{H}$. In Fig. 6b, the conductivity values recovered from the reconstructed conductivity tensor images are compared with the values measured by the impedance analyzer. For the electrolytes #1, #2, #3 and #4, the relative errors of the conductivity values between two methods were 1.1 to 2.4%, 4.4 to 9.2%, 3.39 to 6.52% and 5.26 to 11.48%, respectively, at 10 Hz. For the giant vesicle suspensions in the first and second phantoms, the relative errors of the conductivity values between two methods were 1.7 and 2.13%, respectively, at 10 Hz.

## Discussion

The high-frequency conductivity ($\sigma _\text{H}$) value of the giant vesicle suspension in the first phantom was similar to that of the electrolyte #1. This can be explained from the fact that the electrolyte #1 was used both inside and outside the giant vesicles in the first phantom. Since the thin membranes of the giant vesicles were transparent to 400 MHz currents, the entire giant vesicle suspension appeared as the electrolyte #1 without giant vesicles. This indicates that the high-frequency conductivity does not provide information about the cellular structure.

The high-frequency conductivity ($\sigma _\text{H}$) value of the giant vesicle suspension in the second phantom was between the values of the electrolytes #3 and #4 as shown in Fig. 6a. This can be explained from the fact that high-frequency conductivity ($\sigma _\text{H}$) value of the giant vesicle suspension in the second phantom was a weighted sum of the extracellular conductivity (electrolyte #4) and the intracellular conductivity (electrolyte #3). This supports the basic assumption of the CTI technique in (1). For the pure electrolytes #1, #2 and #4, the extracellular volume fraction $\alpha = 1$ in (1). This results in

9 $$\begin{aligned} \sigma _\text{H} = \sigma _\text{e} = \sigma _\text{L} = \eta d_\text{e}^\text{w} \end{aligned}$$

for pure electrolytes. From the recovered values of $\sigma _\text{H}, \sigma _e, \sigma _\text{L}, \eta$ and $d_\text{e}^\text{w}$, we could confirm that the relation in (9) holds in our experimental data.

In this paper, we used the giant vesicle suspension to physically simulate a cellular structure including both extracellular and intracellular spaces. Controlling viscosity of the electrolytes inside and outside the giant vesicles, we could adjust the extracellular and intracellular conductivity values differently. This allowed us to validate the CTI technique for a more realistic inhomogeneous case compared with the previous case of [11] using the same conductivity value for the extracellular and intracellular spaces. However, the phantom used in this paper was still isotropic since we could not build an anisotropic conductivity phantom using the giant vesicles. Future studies are needed to further validate the CTI technique using an anisotropic conductivity phantom.

For the multi-*b*-value diffusion-weighted imaging part of the experiments, we used 30 gradient directions and 16 *b*-values mainly for the purpose of validating the CTI method without knowing the minimum numbers of gradient directions and *b*-values. This required 124 min of imaging time for each phantom, which is too long for any clinical applications. In our future studies of animal and human subjects, we will reduce the number of gradient directions and b-values and devise better pulse sequences to minimize the imaging time.

The image contrast in CTI is based on ensemble averages of microscopic motions of charge carriers in a structured tissue. Macroscopic CTI image parameters may provide new methods to extract quantitative information about the tissue microstructure and its functions. Clinical applications of CTI may include diagnostic imaging of tumor, ischemia, inflammation, cirrhosis, and other diseases. CTI can also be used for treatment planning by providing patient-specific models for EEG source imaging, transcranial dc stimulation (tDCS), deep brain stimulation (DBS), and electroporation. In general, conductivity tensor images could be useful in many forward problems of bioelectromagnetism. Non-clinical applications in material and food sciences, biology, and chemistry could be also tried.

## Conclusions

The lately developed conductivity tensor imaging (CTI) method reconstructed conductivity tensor images of two conductivity phantom including four electrolytes and two giant vesicle suspensions. The effects of cell density, ion concentration and mobility on the electrical conductivity could be clearly observed from reconstructed conductivity tensor images. The relative errors in the reconstructed conductivity tensor images with respect to the measured conductivity values using an impedance analyzer were in the range of 1.1 to 11.48%. The electrodeless CTI method can be easily implemented in clinical MRI scanners without adding any hardware. Clinical usefulness of the method needs to be verified by future studies of animal and human subjects.

## Methods

### Giant vesicle suspensions

**Fig. 7:**
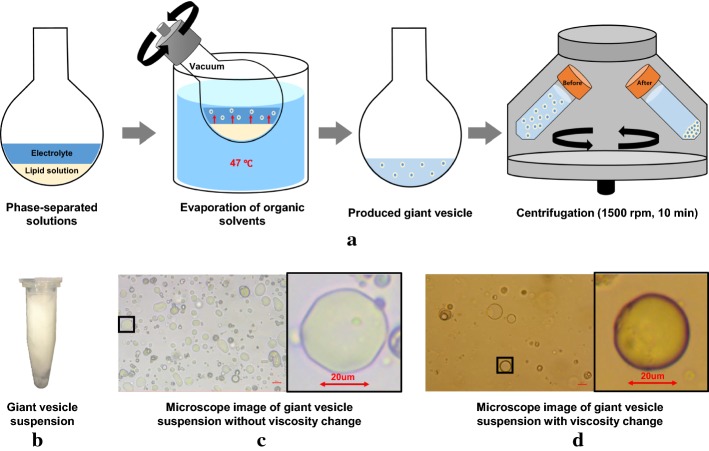
**a** Preparation of giant vesicles.** b** Giant vesicle suspension in a tube.** c**,** d** Microscopic images of two giant vesicle suspensions without and with added hyaluronic acid and polyethylene glycol solution, respectively. The magnified views are marked with the black boxes

Giant vesicles dispersed in an electrolyte were prepared as shown in Fig. 7 [13]. 2 mL of phospholipids (Avanti Polar Lipids, Alabaster, AL, https://avantilipids.com) were dissolved in a round-bottom flask with a chloroform solution of 30 mg/mL concentration under argon atmosphere. The flask was installed to a rotary evaporator (N-1300V-W, EYELA, Tokyo, Japan) to remove organic solvent at $47~^\circ \hbox {C}$ under vacuum with nitrogen trap for 20 min at 10 rpm and then followed by another 20 min at 60 rpm. During evaporation of organic solvent, the phospholipids were assembled to form giant vesicles. The aqueous solution containing the giant vesicles was centrifuged at 1500 rpm for 10 min. The volume fractions of the giant vesicle suspensions were estimated as 50 to 90% from visual observations of their microscopic images. The mean ± standard deviation (SD) of the diameters of the giant vesicles were $13\pm 4.7~\upmu \hbox {m}$.

### Conductivity phantoms

**Fig. 8:**
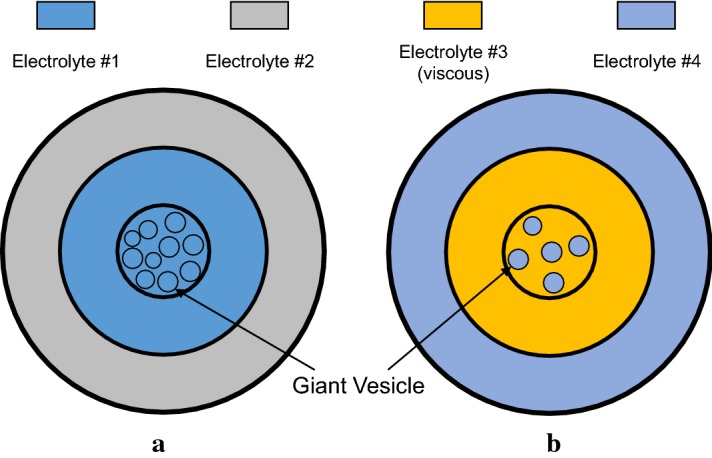
Schematic views of the **a** first and** b** second conductivity phantoms, respectively. The electrolytes #1 and #2 were used in the first phantom and the electrolytes #3 and #4 were used in the second phantom

Figure 8 shows two conductivity phantoms each with three compartments. Two electrolytes were used in the first phantom in Fig. 8a: electrolyte #1 (dark blue) and electrolyte #2 (gray). The electrolyte #1 was an NaCl solution of 7.5 g/L and the electrolyte #2 was a solution with 3.5 g/L NaCl and $1~\hbox {g/L}\; {\text{CuSO}}_4$. The giant vesicles shown in Fig. 8a were filled with the electrolyte #1 and suspended in the same electrolyte #1. The second phantom shown in Fig. 8b contained the electrolytes #3 (yellow) and #4 (light blue). Hyaluronic acid and polyethylene glycol (PEG) solution were added to the electrolyte #3 to increase their viscosity. Polyethylene glycol (PEG) was a water-soluble polymer that reduced mobility of ions and water molecules [14]. Hyaluronic acid in a stable tertiary structure prevented aggregation of giant vesicles in the electrolyte [15]. In the electrolyte #3, 3 g/L of NaCl, 10 g/L of polyethylene glycol (PEG, average Mv  8000) and 2 g/L of hyaluronic acid were added. The electrolyte #4 contained 3 g/L of NaCl only. In the second phantom, the giant vesicles were filled with the electrolyte #4 and immersed in the electrolyte #3.

### Measurements of conductivity spectra

The conductivity spectra of the electrolytes and giant vesicle suspensions used in both phantoms were measured by using an impedance analyzer (SI1260A, AMETEK, UK) with the four-electrode method. The conductivity spectra were obtained in the frequency range of 10 Hz to 3 MHz.

### CTI imaging experiments

**Fig. 9:**
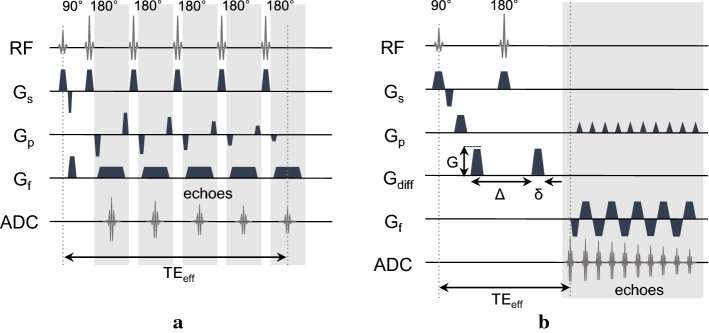
Pulse sequences for conductivity tensor imaging (CTI):** a** multi-echo spin-echo pulse sequence for B1 mapping and **b** spin-echo echo-planar imaging sequence for multi-*b*-value diffusion-weighted imaging

A 9.4-T research MRI scanner (Agilent Technologies, USA) equipped with a single-channel mouse body coil was used. After manual global shimming and acquiring fast localization images, five slices of images were acquired in each imaging sequence with the isotropic resolution of $0.5~\hbox {mm}^3$. The multi-echo spin-echo (MSE) imaging sequence in Fig. 9a was used to acquire B1 phase maps to reconstruct high-frequency conductivity images of the phantoms. The imaging parameters were as follows: repetition time $(T_\text{R})$ = 2200 ms, echo time $(T_\text{E})$ = 22 ms, number of echoes $(N_\text{E})$ = 6, slice thickness = 0.5 mm, number of sampling (NSA) = 5, field-of-view (FOV) = $65\times 65~\hbox {mm}^2$, flip angle = $90^\circ$ and image matrix size ($N_x\times N_y$) = $128\times 128$. The image acquisition time (TA) for each B1 map was about 23 min. The single-shot spin-echo echo-planar (SS-SE-EPI) imaging sequence in Fig. 9b was used for multi-*b*-diffusion-weighted imaging. The number of gradient directions was 30 and 16* b*-values were used for each direction. The imaging parameters were as follows: repetition time $(T_\text{R})$ = 2000 ms, echo time $(T_\text{E})$ = 70 ms, slice thickness = 0.5 mm, number of sampling (NSA) = 2, echo train length (ETL) = 28, field-of-view (FOV) = $65\times 65~\hbox {mm}^2$, flip angle = $90^\circ$ and image matrix size ($N_x\times N_y$) = $128 \times 128$. The chosen* b*-values were 0, 50, 150, 300, 500, 700, 1000, 1400, 1800, 2200, 2600, 3000, 3600, 4000, 4500 and $5000~\hbox {s/mm}^{2}$. With the time interval $\Delta$ = 53.8 ms and duration $\delta$ = 6 ms, the resulting diffusion time ($T_\text{D}$) was ($\Delta -\delta /3$) = 51.8 ms. The imaging time for each phantom was 124 min. For structural imaging, $T_2$-weighted images were also acquired using the multi-slice spin-echo imaging sequence with $T_\text{R}$/$T_\text{E}$ = 3000/80 ms. The imaging time for each $T_2$-weighted image was about 5 min.

### Data processing

Conductivity tensor images of the phantoms were reconstructed using the MRCI toolbox [16] for MRI-based conductivity imaging. The acquired B1 phase images were unwrapped using the PUMA algorithm [17]. Combining all echo signals, average B1 phase images over all acquired echoes were computed [18]. The average B1 phase images were then used to reconstruct high-frequency conductivity images [19]. The acquired diffusion-weighted images were corrected to compensate eddy-current effects and geometrical distortions in the EPI sequence using the ExploreDTI software [20]. The following CTI formula was used for conductivity tensor image reconstructions [10]:

10 $$\begin{aligned} \mathbf{C} = \frac{\alpha \sigma _\text{H}}{\alpha {d_\text{e}^\text{w}} + (1-\alpha ) {d_\text{i}^\text{w}} \beta }\mathbf{D} _\text{e}^\text{w} = \eta \mathbf{D} _\text{e}^\text{w}, \end{aligned}$$

where $\mathbf{C}$ is the conductivity tensor, $\sigma _\text{H}$ is the high-frequency conductivity at the Larmor frequency, $\alpha$ is the extracellular volume fraction, $\beta$ is the ion concentration ratio of intracellular and extracellular spaces, $d_\text{e}^\text{w}$ and $d_\text{i}^\text{w}$ are the extracellular and intracellular water diffusion coefficients, respectively, and $\mathbf{D} _\text{e}^\text{w}$ is the extracellular water diffusion tensor. Extracellular diffusion tensor ($\mathbf{D} _\text{e}^\text{w}$) maps were estimated assuming that water diffusion in the extracellular space was hindered [10, 21]. Throughout the paper, we used the standard Cartesian coordinate system of an MRI scanner, where the *z* axis is along the direction of the main magnetic field of the scanner.

### Estimation of $d_\text{e}^\text{w}$, $d_\text{i}^\text{w}$, $\alpha$ and $\beta$

In this section, we briefly describe the computational methods to estimate the intermediate variables of $d_\text{e}^\text{w}$, $d_\text{i}^\text{w}$, $\alpha$ and $\beta$. More details can be found in [10]. Since the macroscopic voxel includes both extracellular and intracellular spaces, the multi-*b*-value diffusion data can be fitted to a decay curve of multiple exponentials. In this paper, we adopted the following three-compartment model for each voxel [22, 23]:

11 $$\begin{aligned} S_b=S_0\cdot \left( \nu _{\text{ecw}}e^{-bd^{\text{w}}_{\text{ecw}}}+ \nu _{\text{ecm}}e^{-bd^{\text{w}}_{\text{ecm}}}+\nu _{i}e^{-bd^\text{w}_\text{i}}+\nu _0\right) , \end{aligned}$$

where $\nu _{\text{ecw}},\nu _{\text{ecm}}$ and $\nu _\text{i}$ are the volumes and $d^\text{w}_{\text{ecw}},d^\text{w}_{\text{ecm}}$ and $d^\text{w}_\text{i}$ are the water diffusion coefficients of the compartments for the extracellular water, extracellular materials and intracellular space, respectively, with $\nu _0$ denoting an offset value. Note that $S_b$ and $S_0$ are the multi-*b*-value diffusion data for a chosen b-value and $b=0$, respectively. The acquired multi-b-value diffusion data were fitted to (11) for each voxel to compute $\nu _{\text{ecw}},d^{\text{w}}_{\text{ecw}},\nu _{\text{ecm}},d^\text{w}_{\text{ecm}},\nu _\text{i}$ and $d^w_i$ of the voxel. The extracellular volume fraction $\alpha$ and diffusion coefficient $d^w_e$ at each voxel were computed as

12 $$\begin{aligned} \begin{array}{lll} \alpha &{}=&{}\displaystyle \frac{\nu _{\text{ecw}}+\nu _{\text{ecm}}}{\nu _{\text{ecw}}+\nu _{\text{ecm}}+\nu _\text{i}}\\ d^\text{w}_e&{}=&{} \displaystyle \frac{\nu _{\text{ecw}}}{\nu _{\text{ecw}}+\nu _{\text{ecm}}}d^{\text{w}}_{\text{ecw}}+\frac{\nu _{\text{ecm}}}{\nu _{\text{ecw}}+\nu _{\text{ecm}}}d^{\text{w}}_{\text{ecm}}. \end{array} \end{aligned}$$

We set $\beta =1$ in this paper since the ion concentrations inside and outside the giant vesicles were same in the designed phantoms. Note that the conductivity values inside and outside the giant vesicles were, however, set differently by selectively adjusting viscosity of the electrolytes filling the internal and external spaces of the giant vesicles.

## Data Availability

The data that support the findings of this study are available from the corresponding author, [EJW], upon reasonable request.

## Abbreviations

CTI: Conductivity tensor imaging
MRI: Magnetic resonance imaging
MRCI: Magnetic resonance conductivity imaging
DTI: Diffusion tensor imaging
EPI: Echo-planar imaging
PUMA: Phase unwrapping MAx-flow
